# Robust Global Motion Estimation for Video Stabilization Based on Improved K-Means Clustering and Superpixel

**DOI:** 10.3390/s21072505

**Published:** 2021-04-03

**Authors:** Rouwan Wu, Zhiyong Xu, Jianlin Zhang, Lihong Zhang

**Affiliations:** 1 Key Laboratory of Optical Engineering, Institute of Optics and Electronics, Chinese Academy of Sciences, Chengdu 610209, China; wurouwan19@mails.ucas.ac.cn; 2 Institute of Optics and Electronics, Chinese Academy of Sciences, Chengdu 610200, China; jlin@ioe.ac.cn (J.Z.); zhanglihong19@mails.ucas.ac.cn (L.Z.); 3 School of Electronic, Electrical and Communication Engineering, University of Chinese Academy of Sciences, Beijing 100049, China

**Keywords:** video stabilization, global motion estimation, motion vectors, superpixel, simple linear iterative clustering, K-means clustering, feature motion space, video enhancement

## Abstract

Obtaining accurate global motion is a crucial step for video stabilization. This paper proposes a robust and simple method to implement global motion estimation. We don’t extend the framework of 2D video stabilization but add a “plug and play” module to motion estimation based on feature points. Firstly, simple linear iterative clustering (SLIC) pre-segmentation is used to obtain superpixels of the video frame, clustering is performed according to the superpixel centroid motion vector and cluster center with large value is eliminated. Secondly, in order to obtain accurate global motion estimation, an improved K-means clustering is proposed. We match the feature points of the remaining superpixels between two adjacent frames, establish a feature points’ motion vector space, and use improved K-means clustering for clustering. Finally, the richest cluster is being retained, and the global motion is obtained by homography transformation. Our proposed method has been verified on different types of videos and has efficient performance than traditional approaches. The stabilization video has an average improvement of 0.24 in the structural similarity index than the original video and 0.1 higher than the traditional method.

## 1. Introduction

Video filmed on portable cameras frequently suffers from annoying jitters owing to the unsteady motion. Fixed monitoring devices also suffer annoying jitters due to the harsh environment. Video stabilization is the process of improving video quality by removing jitters. This goal can be achieved by using sophisticated sensors and gyroscopes, but they are expensive and inconvenient to deploy. Conversely, digital video stabilization (DVS) does not require additional hardware. It is a convenient and economical solution for different vision tasks.

Obtaining an accurate global motion estimation is a critical step in many vision tasks. In 3D reconstruction, Nachimson et al. [1] used point matching method for global motion estimation; in fall detection, a combination of time-domain and shape detection was used to obtain motion estimation [2]; in action recognition, Wu et al. [3] first used the neural network to obtain the optical flow and used an optimized iterative method to separate it from coarse to fine to obtain a global motion estimation. We mainly research the motion estimation methods used in DVS. According to the different motion models, DVS can be divided into three classes: 2D parameter model methods, 2.5D feature trajectories, and 3D reconstruction methods. The 2D model methods estimate the affines, homographies or bundled homographies between two adjacent frames and smooth the accumulated parameters to remove jitters. They are robust, fast, and effective, but they cannot handle videos with large parallax and large depth transforms, and are also sensitive to processing videos with moving objects and large foreground occlusions. 3D methods reconstruct the 3D camera motion through the structure from motion, and then smooth the motion. Although they are an effective method, they require a huge amount of computation and significantly depend on scene texture. 2.5D methods use feature trajectories to stabilize the video as a combination of the advantages of 2D and 3D methods. They are non-parametric methods that detect the frame’s feature points and use optical flow to track the features. However, the acquired feature points may not fall in the background or disappear, affecting global motion estimation and cause video stabilization failure.

In the 2D methods, Karimi et al. [4] and Xie et al. [5] used a combination of scale-invariant feature transform (SIFT) features and Kanade-Lucas-Tomasi (KLT) trackers to obtain background information, but they were time consuming and could only eliminate single or small object. Shene et al. [6] used speeded up robust features (SURF) cascade and random sample consensus (RANSAC) [7,8] to obtain background information. Although the speed has been improved, it can only eliminate the movement of a single object. Jeon et al. [9] used particles to update the key points, but this method only has good performances for fixed cameras. Wu et al. [10] used K-means clustering to filter the background feature points, but it can only be applied when the background block is larger than the foreground block. Dong et al. [11] used template matching and KLT methods for motion estimation, but inaccurate motion estimation will occur when there is foreground occlusion. In the 2.5D method, Koh et al. [12] used the K-means clustering to filter the feature points’ trajectories, which has a good effect, but it takes a long time and there is a phenomenon of instability due to the disappearance of the trajectory. Ma et al. [13] set different weights for the background and foreground feature trajectories to stabilize the video. Although the method is effective, it also takes a long time and requires lots of memories. Zhao et al. [14] also set penalty factors with different weights for background and foreground feature trajectories for video stabilization but his method is only for traffic videos.

The algorithms of 2D methods are robust and practical but are hard to attack the unstable video with multi objects and large occlusion. The algorithms of 2.5D methods are effective but take a long time. Motivated by the limits of the current 2D methods, we propose a simple and robust algorithm to obtain an accurate global motion estimation. Our method is inspired by Wu et al. [10] and Koh et al. [12]. We improve motion estimation based on feature points in the 2D method. Firstly, we adaptively do simple linear iterative clustering (SLIC) segmentation on the adjacent two frames according to the video size and eliminate the superpixels, whose cluster centers’ absolute values are large. Then, the feature point detection is performed on the remaining superpixels. The feature motion vector space is established according to the matching feature points’ Euclidean distance, and the improved K-means clustering is adopted to eliminate the local motion again. Finally, accurate global motion estimation is obtained, and the global motion is retained from coarse-to-fine. We use thorough experiments to demonstrate that our method outperforms the work of Wu et al. [10] in most cases. The main contribution of this paper can be summarized into the following three aspects.

- We first introduce superpixels into the video stabilization, which enables our approach to share higher efficiency and robustness over existing traditional 2D methods in the global motion estimation step. Besides, our method has an average structural similarity of 0.1 higher than that of the traditional 2D stabilization methods among the different types of videos in the public video stabilization dataset.
- We propose a simple and “plug and play” module that can obtain accurate global motion estimation. It can be directly used in the motion estimation step based on feature point stabilization.
- We improve the K-means clustering, which enables the initial point even distribution and adaptive K. By combining superpixels and improved K-means clustering, we perform a coarse-to-fine elimination of local motion, which overcomes the main challenge of 2D traditional video stabilization—the stabilization of multi-object and large foreground occlusions videos.

The paper is organized as follows—Section 2 briefly presents the related work of video stabilization. Section 3 proposes a global motion vector estimation method based on the combination of improved K-means clustering and SLIC. We evaluate the proposed approach in Section 4 and Section 5 concludes the paper.

## 2. Related Work

**Figure 1:**
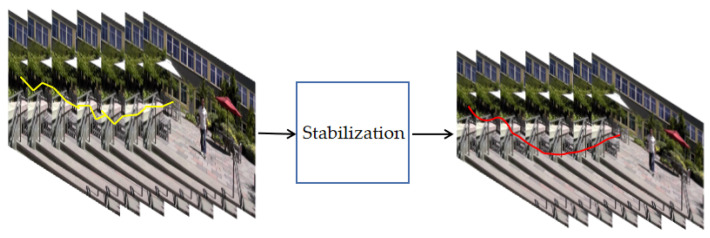
Motion accumulation of unstabilized video and stabilized video.

Video stabilization can be roughly divided into 2D, 2.5D, and 3D methods. We will briefly review it in the following. The result of video stabilization can be illustrated in Figure 1. The yellow line figure represents the global camera path of the jitter video, which has high-frequency noise and is not smooth. The global camera path of the figure with the red line is obtained by stabilizing the image, the path is smoother than before, but the image’s size is also reduced accordingly.

The 2D methods use an affine or a homography matrix to represent the global motion of two adjacent frames. Xu et al. [15] used oriented features from accelerated segment test (FAST) and rotated binary robust independent elementary features (BRIEF) detection operator (ORB) and affine matrix to estimate two adjacent frames. Although the speed of motion estimation is very fast, it can only handle shaky videos with distant and static backgrounds. Shene et al. [6] used the combination of SURF and RANSAC to accurately match the feature points, and used the homography matrix to represent the motion of two adjacent frames. It uses a matrix with more parameters, but perform well on video with a single object. Cheng et al. [16] used a method of combining feature points and improved cascade parameters to estimate the motion of two adjacent frames. Although the model using a matrix is robust and effective to a single plane, it does not solve large parallax and multi-plane. In order to solve this problem, Liu et al. [17] first proposed a method using the bundled camera path. They divide each frame into regular small grids and perform homography calculation and accumulation optimization for each small grid. Following methods for video stabilization using a bundled camera path are also proposed [18]. Although it is more effective than using a single matrix, it takes longer and cannot process videos with large moving objects. In order to satisfy real-time and accuracy, Dong et al. [11] proposed to use three frames of trajectory to predict a homography matrix. Lim et al. [19] proposed an algorithm to tackle the problem of real-time video stabilization for unmanned aerial vehicles (UAVs), where they designed an affine model for the global motion of UAV and employed the combination optical flow and feature point. Hu et al. [20] also proposed a method to achieve real-time video stabilization. However, it still cannot solve the influence of multi-object motion and foreground occlusion on global motion estimation. With the popularity of deep learning, there are also some video stabilization methods based on deep learning. Input stabilized and jitter video to the network, and output a homography matrix to the network [21]. The objective function does not consider the effects of multi-object and parallax, so it is only effective for a single object or background shaky video. Yu et al. [22,23] used neural networks to estimate optical flow to achieve pixel-level video stabilization. But this method is mainly for selfie type videos. We also attribute this method to 2D video stabilization. Although the deep learning method has a good effect on DVS, its portability and real-time performance are not as good as traditional 2D methods.

The 2.5D methods generally store and smooth the feature trajectory. Lee et al. [24] was the first to apply the feature point trajectory to video stabilization research. First, a set of trajectories were collected, and using curve fitting to smooth the trajectory. It controls the cropping rate of the stabilized video, but does not consider multi-object motion and foreground occlusion. Liu et al. [25] model the trajectory matrix of the collected features, perform low-rank decomposition of the matrix and then perform smoothing operations such as curve fitting in the low-dimensional space. Although it can handle parallax and has a good stabilization effect, it is mainly dependent on feature points and long-term tracking. Once the feature points disappear or the trajectory is too short, the video stabilization will fail. In order to solve the problem of trajectory length, Koh et al. [12] used a low-rank matrix method to improve and enhance the trajectory and eliminated the object feature points through a clustering method. Liu et al. [26] used a dense optical flow method to estimate pixels’ motion, solve the problem of dependence on feature points, and filter out the moving object pixels through the histogram iteration of the amount of pixel motion. Ma et al. [13] introduced the idea of grids into the trajectory of feature points, performed adaptive weight calculation on the collected trajectories to obtain the background trajectory and smoothed to obtain a stable video. Although the 2.5D method has a better video stabilization effect and the ability to filter out motion feature points than the 2D method, it is more time-consuming and dependent on video quality than the 2D method.

The 3D methods need to reconstruct the real motion of the camera and then smooth it. The earliest Buehler et al. [27] used image rendering for non-metric reconstruction. Because of the proposal of structure-from-motion (sfm), Zhao et al. [28] introduced sfm into 3D video stabilization and performed 3D reconstruction of the collected characteristic motion. However, this method is very slow and sensitive to parallax changes. The video stabilization effect depends heavily on video quality. In order to reduce the influence of distortion on the original information of the video, Liu et al. [29] introduced content-persevering into the video stabilization and adopted the “as-rigid-as-possible” [30] idea to transform the video stabilization. Zhou et al. [31] added plane constraints to this system to reduce video distortion. Liu et al. [32] also used a depth camera to study video stabilization. Besides, Liu et al. [33] also conducted comprehensive research on the subspace method and applying it to stereoscopic video stabilization. Although the 3D video stabilization method can produce the most satisfactory visual results, the method relies heavily on robust feature trajectories. In practical applications, long feature trajectories are complicated to obtain. Also, this method takes longer and requires more memory.

Some researches presented novel global motion estimation methods; however, most of them are based on the 2.5D model, and it is time-consuming. Liu et al [34] proposed a novel DVS method based on MeshFlow, using two median filters from coarse-to-fine to obtain the global motion optical flow. Although the speed has been improved, it cannot handle the effects of large foreground occlusion and multiple objects on the global optical flow. Dong et al. [10] proposed combining block and three-frame trajectory to perform global motion estimation, but multi-object motion video stabilization is still not robust. Wu et al. [11] used K-means clustering in motion estiomation step but they can only process well on videos with background blocks larger than the object blocks. We designed a coarse-to-fine global motion estimation method to achieve video stabilization of multi-object motion and large foreground occlusion videos.

## 3. Robust Global Motion Estimation

**Figure 2:**
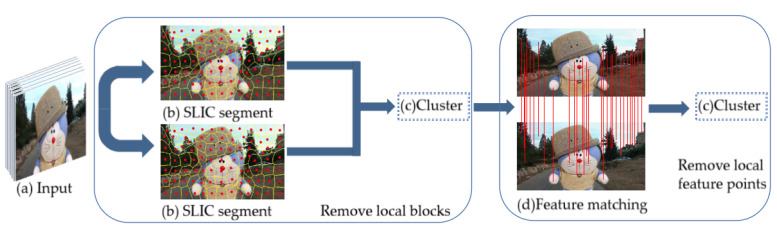
Our proposed method pipeline: the video input (**a**) is partitioned into the simple linear iterative clustering (SLIC) segment (**b**). The superpixel’s centroid motion is clustered, and feature points match between adjacent frames (**d**). The adaptive K-means clustering is applied to remove local feature points.

Our proposed video stabilization is the first to introduce superpixels into the video frame and combine SLIC and K-means clustering to obtain accurate global motion estimation. Figure 2 shows the proposed method’s pipeline, shows the rough steps of motion estimation. In the following, first, we will introduce how to roughly remove local motion blocks, then how to remove local motion feature points accurately, and finally, we show how to combine the proposed method with the existing traditional 2D method based on feature point.

### 3.1. Local Motion Block Removal

In the video, the background and the object are usually in motion, and the combination of block and frame difference is not effective in filtering local motion blocks. Therefore, we use superpixel to replace the block, and cluster the centroid motion of the superpixels to eliminate local motion blocks.

**Figure 3:**
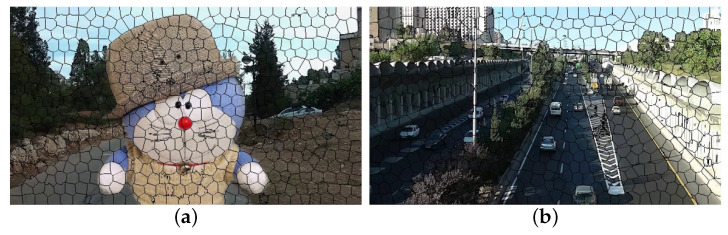
The result of superpixel in two different types of videos: (**a**) large foreground occlusions, (**b**) multi-object.

Superpixel is an image segmentation technology proposed and developed by Ren et al. [35]. It refers to an irregular pixel block with specific visual significance composed of adjacent pixels with similar texture, color, brightness and other characteristics. It uses the similarity of features between pixels to group pixels and replaces a large number of pixels with a small number of superpixels to express image features, which significantly reduces the complexity of image post-processing. Therefore, the background can be clustered into one category, and the object can be clustered into one category more accurately, which is convenient for subsequent processing. Figure 3 shows the result of superpixel segmentation.

To eliminate local motion blocks faster and more accurately, we first use SLIC [36] to segment the image and calculate the amount of motion based on the obtained superpixel centroid coordinates of two adjacent frames with the same label, and establish a motion vector space. Assume the image only contains the object, and the background motion is usually less than object motion. We set cluster K=2, and superpixels with a large cluster center value are eliminated to obtain the coarse background image.

Before performing SLIC segmentation on the image, the color image needs to be converted into a 5-dimensional feature vector in the Lab color space and XY coordinates, where $L^{*}$ represents the brightness, $a^{*}$ represents the range from magenta to green, and $b^{*}$ represents the range from yellow to blue range. First, the number of superpixels needs to be set. Through our experiments, the adaptive selection of the number of superpixels is shown in Equation (1).

(1) $$K_{s}=\frac{100*w*h}{640*360}.$$

Assuming that the image has a total of *N* pixels, pre-segmented into $K_{s}$ pixels of the same size, then the size of each superpixel is $N/K_{s}$, and the distance between adjacent cluster centers is $S=\sqrt{N/K_{s}}$. Then reselect the cluster center in the 3∗3 area of the seed point, assign a class label to each pixel, and measure the distance of the pixel that meets the search range of 2S*2S. The calculation Equation is shown in (2). Where i represents the *i*th pixel, j represents the cluster center of the *j*th category, $d_{c}$ represents the color distance, $d_{s}$ represents the spatial distance, $N_{s}$ is the maximum spatial distance within the class, and $N_{c}$ is the maximum color distance. Because $N_{c}$ cannot be determined, it is used *m* represents the relative importance of space and pixel color. We sets m = 30. The distance metric can be written as shown in Equation (3). According to the above steps, iterate continuously until the cluster center no longer changes. Generally, the number of iterations is 10.

(2) $$\begin{array}{cccc} \; & d_{c} & = & \left\{\begin{array}{cc} \sqrt{{\left(l_{j}-l_{i}\right)}^{2}+{\left(a_{j}-a_{i}\right)}^{2}+{\left(b_{j}-b_{i}\right)}^{2}} & \;\left(\mathrm{color}\right)\;\\ \sqrt{{\left(l_{j}-l_{i}\right)}^{2}} & \;\left(\mathrm{gray}\right)\; \end{array}\right.\\ \; & d_{s} & = & \sqrt{{\left(x_{j}-x_{i}\right)}^{2}+\left(y_{j}-y_{i}\right)}\\ \; & D^{\prime } & = & \sqrt{{\left(\frac{d_{c}}{N_{c}}\right)}^{2}+{\left(\frac{d_{s}}{N_{s}}\right)}^{2}} \end{array}$$

(3) $$D^{\prime }=\sqrt{{\left(\frac{d_{c}}{m}\right)}^{2}+{\left(\frac{d_{s}}{S}\right)}^{2}}.$$

When the superpixels of adjacent frames are obtained, we compute the Euclidean distance between the centroid coordinates of the superpixels with the same label to obtain the motion of the superpixel and establish a coordinate space based on the motion. Then take the cluster with K=2, and remove the superpixel block with a large cluster center. The superpixel motion vector of adjacent frames can be expressed as Equations (4)–(6), Where $K_{s}$ represents the number of superpixels, and $l_{i}^{x^{\prime }}$ and $l_{i}^{x}$ represent the centroid’s horizontal coordinates of the superpixels with the same label in adjacent frames. Similarly, $l_{i}^{y}$ and $l_{i}^{y^{\prime }}$ represent the centroid’s vertical coordinates of the corresponding superpixels. $M_{c_{i}}$ represents the coordinate of the corresponding superpixel in the 2D motion vector space.

(4) $$M_{c_{i}}\left(m_{c_{i}^{x}},m_{c_{i}^{y}}\right),\;\;\mathrm{where}\;i=1,2,\ldots K_{s}$$

(5) $$m_{c_{i}^{x}}=\sqrt{{\left(l_{i}^{x}-l_{i}^{x^{\prime }}\right)}^{2}}$$

(6) $$m_{c_{i}^{y}}=\sqrt{{\left(l_{i}^{y}-l_{i}^{y^{\prime }}\right)}^{2}}.$$

**Figure 4:**
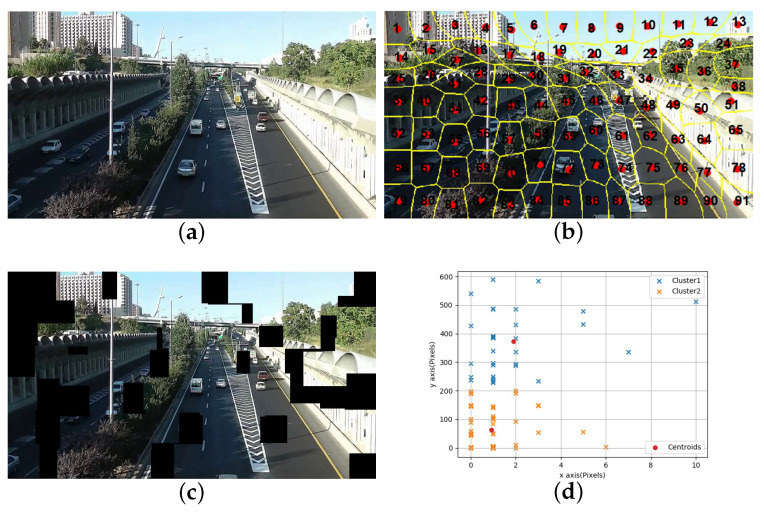
Experimental results of using superpixels to eliminate local motion blocks: (**a**) original image, (**b**) superpixel image, (**c**) the proposed method of local motion block removal, (**d**) motion vectors cluster.

Figure 4 shows the result of using SLIC to segment and remove the local motion blocks for the t-th frame of the shaky video. Figure 4a shows the original image of the t-th frame, and Figure 4b shows the labeled superpixel image after SLIC segmentation, and Figure 4c shows the image after the motion block is removed by the proposed method, and Figure 4d is the centroid motion vector cluster map, with red dots represents the cluster center. Because there is the movement of the background and the object and the sudden shaking in the video, black blocks will appear in both the background and the object.

### 3.2. Local Motion Feature Removal

Although the potential local motion blocks are eliminated, two types of mismatches will inevitably occur when matching feature points. The first is the mismatch of feature points in two adjacent frames, and the second is that the matching points fall on the object instead of the background due to the existence of local motion. RANSAC can solve the first mismatch, and the second mismatch has no effective solution. K-means clustering is a practical and simple method, which is often used in image processing. Khan et al. [37] proposed adaptive K-means clustering initialization parameters based on the distribution of gray histograms. The difference between Khan’s method is that we improve the K-means clustering based on the motion vector’s difference and the background and foreground’s motion characteristics. Improved K-means clustering is used to eliminate the second type of mismatched points, and then homography transformation is computed from the retained global feature points. This method was inspired by Koh [12]. They processed the motion trajectory to obtain the trajectory velocity, clustered it, and obtained the global motion feature trajectory.

In the step of detecting and matching feature points, we use SURF features [38] to perform corresponding experiments. Among the matching feature points in two adjacent frames, the motion vector of the matching point is calculated to establish a 2D motion vector space, as shown in Equations (7)–(9), where *n* represents the number of matching feature points, $f^{x_{i}}$ and $f^{x_{i}^{\prime }}$ represent the horizontal coordinates of the matching feature points in two adjacent frames, $f^{x_{i}}$ and $f^{y_{i}^{\prime }}$ represent the vertical coordinates of the matching feature points in two adjacent frames, and $M_{p}$ represents the feature motion space established based on the motion vectors of the matching feature points.

(7) $$M_{p_{i}}\left(m_{p_{i}^{x}},m_{p_{i}^{y}}\right),\;\mathrm{where}\;i=1,2,\ldots n$$

(8) $$m_{p_{i}^{x}}=\sqrt{{\left(f_{i}^{x}-f_{i}^{x^{\prime }}\right)}^{2}}$$

(9) $$m_{p_{i}^{y}}=\sqrt{{\left(f_{i}^{y}-f_{i}^{y^{\prime }}\right)}^{2}}.$$

To get as accurate a motion estimation as possible, we make two improvements to K-means. The first is to make the initial cluster centers as evenly distributed as possible, and the second is to adjust the value of *K* adaptively. There are a total of $\left\{M_{p_{1}},M_{p_{2}},\ldots M_{p_{n}}\right\}$ points in the motion vector space we have established, which need to be clustered into *K* categories. The initial cluster centers $\left\{C_{1},C_{2},\ldots ,C_{K}\right\}$ are calculated in Equations (10)–(12),

(10) $$d_{x}=\max_{i,j=1,\ldots ,n}\left(m_{p_{i}^{x}}-m_{p_{j}^{x}}\right),d_{y}=\max_{i,j=1,\ldots ,n}\left(m_{p_{i}^{y}}-m_{p_{j}^{y}}\right)$$

(11) $$g=\arg\min_{i}\left(m_{p_{i}^{x}},m_{p_{i}^{y}}\right)$$

(12) $$\left\{\begin{array}{c} C_{1}=\left(m_{p_{g}^{x}}+\frac{dx}{K},m_{p_{g}^{y}}+\frac{dy}{K}\right)\\ C_{2}=\left(m_{p_{g}^{x}}+\frac{2dx}{K},m_{p_{g}^{y}}+\frac{2dy}{K}\right)\\ \;\ldots \\ C_{K}=\left(m_{p_{g}^{x}}+dx,m_{p_{g}^{y}}+dy\right), \end{array}\right.$$

where $d_{x}$ and $d_{y}$ represent the maximum horizontal distance and the maximum vertical distance in the feature motion space, *g* represents the index of the matching point closest to the origin of the feature motion space.

In order to find the optimal cluster *K*, we need to define a judgment factor *a*, using the intra cost within each cluster and the inter cost between cluster. We define a dissimilarity distance between $C_{1}$ and $C_{K}$, as shown in Equation (13).

(13) $$d\left(C_{K},C_{l}\right)=\frac{\sum_{i\in C_{K}}{∥M_{p_{i}}-C_{l}∥}^{2}}{\left|C_{K}\right|},$$

where $|C_{K}|$ is the number of cluster points, $C_{l}$ represents the cluster center point, $M_{p_{i}}$ represents the coordinate point belonging to cluster *K*, and *d* is the average of the differences from the point included in cluster *K* to the cluster center of cluster *l*. We then define the intra cost and the inter cost as shown in Equation (14).

(14) $$\left\{\begin{array}{c} Intra\left(C_{1},C_{2},\ldots ,C_{K}\right)=\frac{\sum_{i=1}^{K}d\left(C_{i},C_{i}\right)}{K}\\ Inter\left(C_{1},C_{2},\ldots ,C_{K}\right)=\frac{\sum_{i=1}^{K}\sum_{l=1,l\neq i}^{K}d\left(C_{i},C_{l}\right)}{K(K-1)}. \end{array}\right.$$

Intra represents the average similarity between the same class and the cluster centers, and Inter represents the average dissimilarity between the cluster centers of different classes.

For efficient clustering, we can select the optimal number $K^{*}$ with the minimum ratio of the intra cost to the inter cost, as shown in Equation (15).

(15) $$\left\{\begin{array}{c} a=\frac{\mathrm{Intra}\left(C_{1},C_{2},\ldots ,C_{K}\right)}{\mathrm{lnter}\left(C_{1},C_{2},\ldots ,C_{K}\right)}\\ K^{*}=\underset{K\in \{2,\ldots ,5\}}{\mathrm{argmin}}a. \end{array}\right.$$

**Figure 5:**
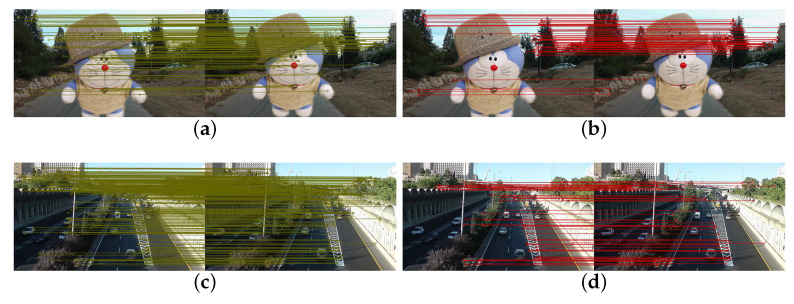
Feature point matching: (**a**) using RANSAC in large object occlusion shaky video, (**b**) proposed method in large object occlusion shaky video, (**c**) using RANSAC in multiply objects shaky video, (**d**) proposed method in multiply objects shaky video.

Figure 5 shows the result of removing local feature points using our proposed method. Figure 5a shows using RANSAC to eliminate mismatches in large foreground occlusions frame. Figure 5b shows using our proposed method to eliminate mismatches in large object occlusion frame. Figure 5c shows using RANSAC to eliminate mismatches in multiple objects frame. Figure 5d shows using our proposed method to eliminate mismatches in multiple objects frame. Both the yellow and red lines indicate the connection of the matching points.

**Figure 6:**
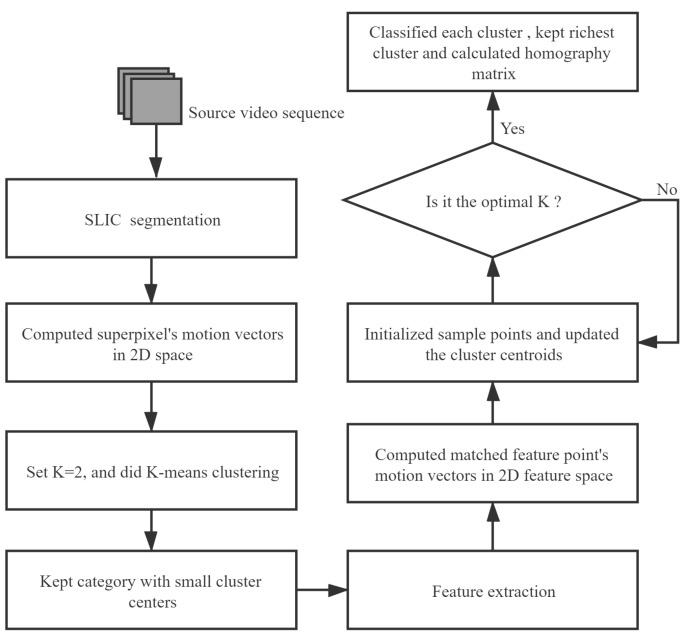
The flowchart of our proposed method.

From the analysis above and the overall framework in Figure 2, the proposed algorithm’s flowchart is shown in Figure 6.

The traditional 2D method of using feature points for motion estimation generally consists of three steps: feature point extraction, RANSAC to eliminate mismatches, and calculation of the transformation matrix. We only need to change the eliminate mismatches step to our proposed method to get accurate motion estimation.

## 4. Experimental Results and Discussion

In this section, first, we will compare the proposed method with typical methods that use feature points and a single matrix for video stabilization. The proposed method improves the video quality by computing accurate global motion using SLIC segment and K-means clustering. To verify the effectiveness of our method, we present a set of comparative experiments. Next, we will show the proposed method’s performances on shaky videos with large object occlusion and multi-object motion. Similarly, we prove the effectiveness of our method through analysis with the traditional 2D methods.

### 4.1. Comparison of Different Video Stabilization Methods Based Feature Points

**Figure 7:**
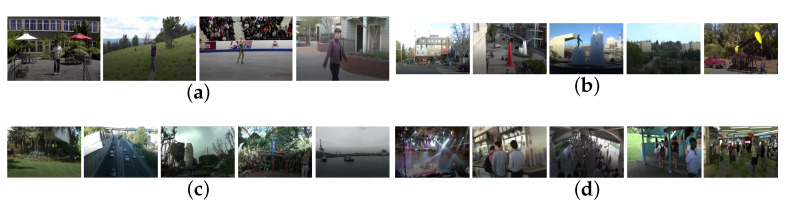
The tested image sequence: (**a**) shaky videos with single object, (**b**) only background shaking videos, (**c**) shaky videos with zooming, (**d**) shaky videos with crowds.

**Figure 8:**
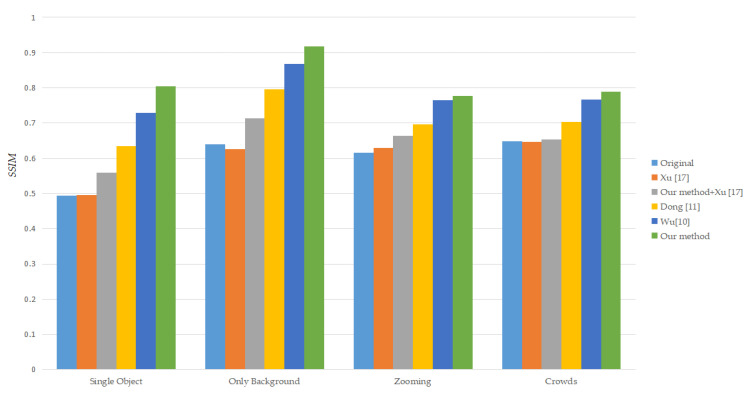
Average SSIM of different methods in four groups shaky videos.

In order to prove that our proposed method is more effective than the previous video stabilization method based feature points, we used four groups of 19 videos in total [39]. Moreover, our proposed method is a “plug and play” module, so we add it to the existing method for verification its effectiveness. We use the average structural similarity as the criterion. The closer the value is to 1, the better effective method. The average SSIM [40] of each group of videos is shown in Table 1, Table 2, Table 3 and Table 4. The first frames of these unstabilized image sequences are shown in Figure 7. The average SSIM of different methods for different groups is shown in Figure 8 and Table 5.

As shown in Table 1, Table 2, Table 3 and Table 4, we compare our method with two existing 2D approaches in four different group videos. They are Xu [17], Dong [11], and Wu [10]. Besides, we add our method to Xu [17] to verify its effect “plug and play”. Before calculating the transformation matrix, add our proposed method. We implement the methods of Xu [17] based on our module and Wu [10] by ourselves. The code of initial Xu [17] is found at https://github.com/francocurotto/Video-Stabilization (accessed on 2 April 2021). Thanks to the authors of Dong [11], they provide the binary implementation of their approaches at http://Real-timeDVS.blogspot.com/ (accessed on 2 April 2021).

Xu [17] uses RANSAC and Dong [11] uses the combination of three frames feature point trajectory and RANSAC to eliminate local motion. Table 1, Table 2, Table 3 and Table 4 show different method’s average SSIM in four group shaky videos. Our proposed method can obtain more stable video and accurate global motion under the same feature extraction method and filter by comparing the first, fifth, and sixth rows of each table. By comparing the second and third rows of each table, we can find that adding our proposed method to the existing methods can improve video stabilization quality. In Figure 8, we use bar graph to illustrate the data in Table 1, Table 2, Table 3 and Table 4. The height of each bar is the different method’s average SSIM in a group. It can be seen that our method is effective than other methods that use a single matrix for motion estimation. Compared with the average SSIM of the original video, our method has an average improvement of 0.24. On the other hand, it also shows that use a combination of SLIC and improved K-means clustering for motion estimation, which can get a more accurate global motion vector and eliminate redundant local motion.

### 4.2. The Results of Large Foreground Occlusion’s and Multi-Object Motion’s Stabilized Video

**Figure 9:**
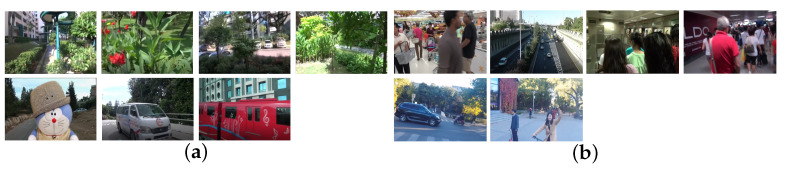
The tested image group: (**a**) shaky videos with large foreground, (**b**) shaky videos with multi-object.

These data come from different public data sets, which can be obtained publicly on the website [39,41]. We show our proposed method’s effectiveness by comparing the average SSIM of the original video, and other based feature point method video. The first frames of these unstabilized video sequences are shown in Figure 9. The average SSIM of each group of videos is shown in Table 6 and Table 7:

In Table 6, the average SSIM of video 4 before and after video stabilization does not improve much because the video has a certain parallax. Our proposed method uses a single matrix for motion estimation, which has certain limitations in this type of video. In Table 7, the average SSIM of video 6 before and after video stabilization is not improved much, because the video has motion blur, which leads to inaccurate positioning of feature points, which also leads to the failure of motion estimation. In addition to these two videos, we can know by comparing other video results in Table 6 and Table 7, when we use our proposed method to process jittery videos with large foreground occlusion and multi-target motion, the average SSIM can be increased and the viewing experience can be improved. Compared with the other two methods based on feature points, our method has a greater improvement in average SSIM.

**Figure 10:**
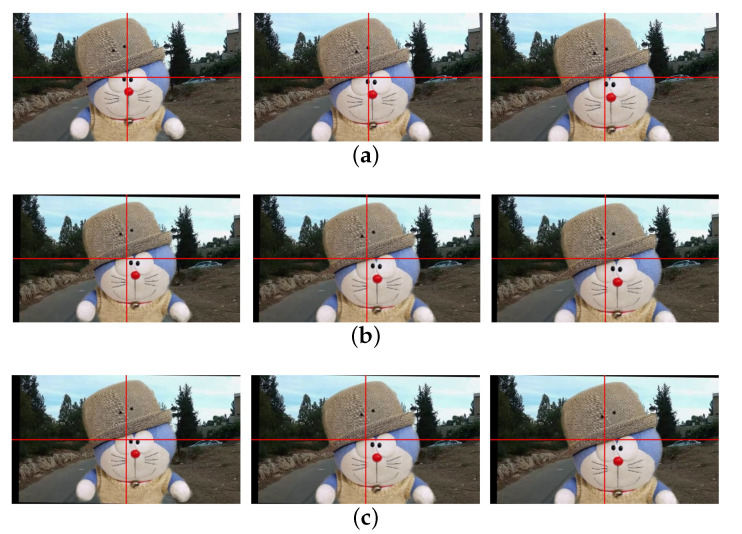
Experiment results of different video stabilization methods: (**a**) shaky video frames with large foreground occlusion (66th, 67th, and 68th frames), (**b**) the stabilized video of [10], (**c**) our method.

We use the same filter to filter the obtained motion vector and obtain a stable video through motion compensation. Figure 10a shows the three original frames, and Figure 10b shows the stabilized results using the combination of K-means clustering and RANSAC [10]. As shown in Figure 10c, the proposed method successfully obtains stabilized video with the less black region. It shows that our method can better reduce the influence of local motion on global motion estimation.

**Figure 11:**
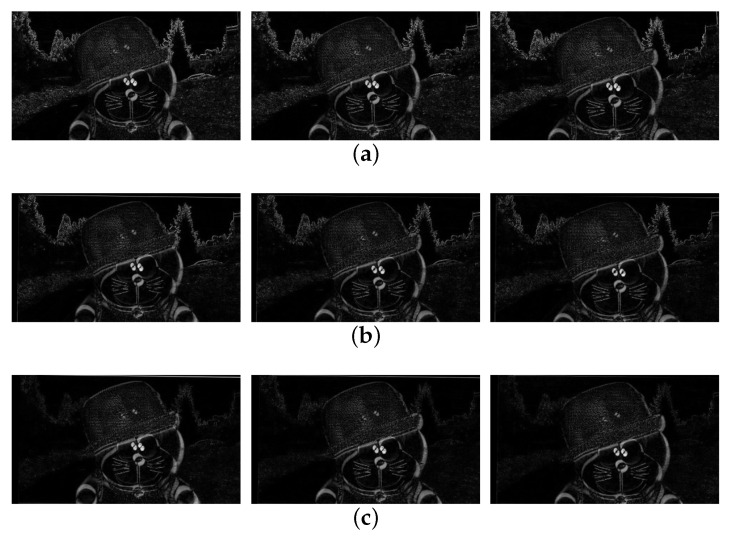
Experiment results: (**a**) differences of original video (66th, 67th, and 68th frames), (**b**) the stablized video of [10] (66th, 67th, 68th frames), (**c**) our stabilized video (66th, 67th, and 68th frames).

Figure 11a shows the difference map of three pairs of original frames (66,67), (67,68), (68,69), and Figure 11c shows the difference map of three pairs of stabilized frames (66,67), (67,68), (68,69). Through the comparison, we can know that our method removes the jitter on the background very well and retains the subjective motion of the moving object.

**Figure 12:**
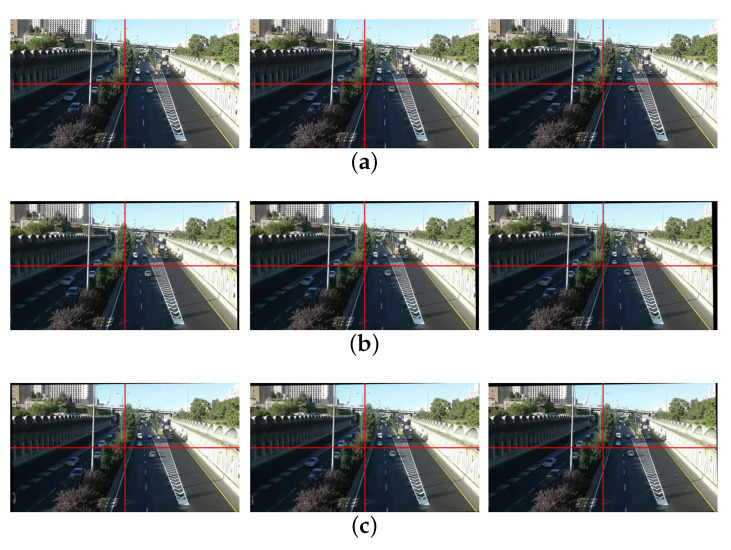
Experiment results of different video stabilization methods: (**a**) shaky video frames with multi-object motion (129th, 130th, and 131th frames), (**b**) the stabilized video of [10], (**c**) our method.

Figure 12 shows the video stabilization effect of the different methods in a multi-object motion video. Figure 12a is the original frame, Figure 12b is the result of video stabilization using the combination of K-means clustering and RANSAC [10], and Figure 12c is the result of our proposed method. By comparison, our method has fewer black region.

**Figure 13:**
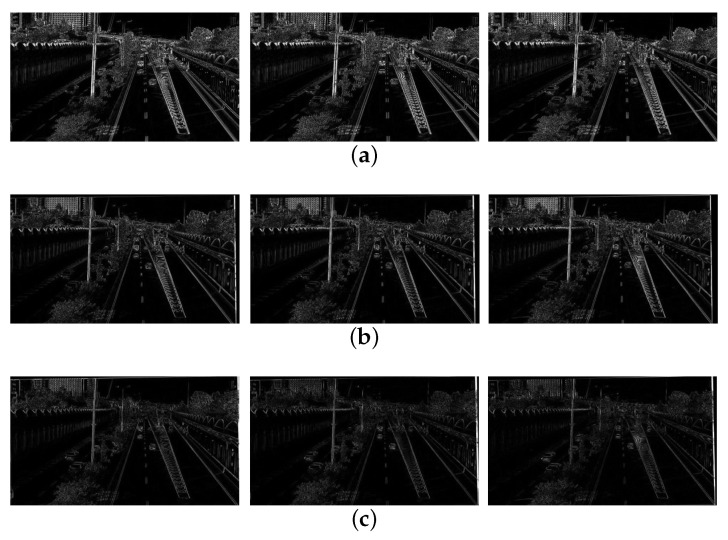
Experiment results: (**a**) differences of original video (129th, 130th, and 131st frames), (**b**) the stabilized video of [10] (129th, 130th, and 131st frames), (**c**) our stabilized video (129th, 130th, and 131st frames).

Figure 13 shows the difference results. Figure 13a shows the difference of the original shaky frame (129,130), (130,131), (131,132), Figure 13b shows the difference of the stabilized video frame (129,130) ), (130,131), (131,132) by [10], and Figure 13c shows the difference of the stabilized video frame (129,130) ), (130,131), (131,132) by our method. Through the comparison of the images of each frame, it can be found that the differential image by our proposed method is smoother than the result by [10].

Through the display in Figure 10, Figure 11, Figure 12 and Figure 13, we can know that when performing motion estimation on shaky videos with large foreground occlusion and multi-object motion, the combination of SLIC and improved K-means clustering can obtain a more accurate global motion estimation than Wu [10]. The stabilized results using the proposed method can be found in the supplementary video, and the supplementary video also shows the different video stabilization’s results based on 2D feature detection.

### 4.3. Discussion

Among the methods that use feature points for motion estimation, most of them use RANSAC to eliminate mismatches [11,17] and some use K-means clustering to cluster feature points [10]. The K-means clustering method has accurate motion estimation for shaky videos when the background larger than the foreground. Methods such as RANSAC can only eliminate mismatched points, and cannot solve the impact of local motion on global motion estimation. Our method based a combination of SLIC segment and improved K-means clustering can not only obtain global motion estimation in common shaky videos, but also perform global motion estimation on shaky videos with large foreground occlusion and multi-target motion. Of course, our method also has some shortcoming: (1) as shown in Table 6, we can find the average SSIM of video 4 has little improvement because the video has large parallax, which means our method can only process planar video; (2) as shown in Table 7, video 6 average SSIM also has little improvement because our method is difficult to process shaky videos with motion blur.

To our knowledge, we are the first to use superpixels for video stabilization. Although it is more effective than the existing method of using 2D feature detection for motion estimation, it also faces parallax and motion blur. This is also a problem faced by many video stabilization methods.

## 5. Conclusions and Future Work

This paper proposes a robust and simple method to address the problem of obtaining accurate global motion estimation in video stabilization. We show how to combine SLIC segment and improved K-means clustering to remove local motion from coarse to fine through the analysis of the motion vector. We show how to add this module to existing 2D motion estimation based feature matching, which is usually ignored in the previous 2D approach.

Our study shows that our proposed method can obtain stabilized videos that are better than previous 2D approaches in a measurement that considers average SSIM. In addition, our method also has a useful video stabilization effect on shaking videos with large foreground occlusion and multi-object motion. By stabilizing different types of shaking videos, we find that the proposed method can be used for various video applications, including portable shooting equipment, video surveillance systems, and many vehicle imaging systems.

In our implementation, the number of superpixels is set adaptively under the video frame size, the centroid motion of the superpixels is calculated, and the motion space is established. Then setting K to 2 is used for coarse clustering, and blocks with a large amount of motion are eliminated. Feature points detect on the remaining superpixels, calculate the matching feature points’ movement, establish the feature movement space, use the improved K-means clustering method to cluster the feature movement, and save the points with a large number of clusters to obtain accurate background features point and perform motion estimation. Our proposed method’s two drawbacks are that we use a single matrix to estimate global motion, which is not suited for processing shaky videos with parallax. And our method is based on feature detection. There will be feature point positioning errors when the video contains motion blur. For strict real-time application, this may imply adaptive frame’s SLIC segment, not each frame’s segment. So in future works, we will focus on those challenging situations. In addition, a better way to apply superpixels to the field of video stabilization is also future work.

## Figures and Tables

**Table 1:** SSIM Comparison of different methods for shaky videos with single object.

	Video 1	Video 2	Video 3	Video 4
Original	0.3565	0.4536	0.6734	0.4944
Xu [17]	0.3333	0.4637	0.6831	0.5054
Our method + Xu [17]	0.462	0.5107	0.6961	0.5661
Dong [11]	0.6144	0.6028	0.7065	0.6113
Wu [10]	0.7367	0.7292	0.7511	0.6985
Our method	0.8406	0.7997	0.8102	0.7657

**Table 2:** SSIM Comparison of different methods for only background shaking videos.

	Video 1	Video 2	Video 3	Video 4	Video 5
Original	0.4910	0.5329	0.8051	0.8069	0.5639
Xu [17]	0.4540	0.5353	0.7777	0.7987	0.5677
Our method + Xu [17]	0.6009	0.6555	0.8248	0.8185	0.6704
Dong [11]	0.7285	0.7549	0.8962	0.8543	0.7449
Wu [10]	0.8355	0.8407	0.9462	0.8896	0.8304
Our method	0.9218	0.9297	0.9686	0.8781	0.8922

**Table 3:** SSIM Comparison of different methods for shaky videos with zooming.

	Video 1	Video 2	Video 3	Video 4	Video 5
Original	0.6696	0.4870	0.7239	0.4699	0.7313
Xu [17]	0.6937	0.5066	0.7407	0.4908	0.7123
Our method + Xu [17]	0.7484	0.5672	0.7698	0.52	0.7154
Dong [11]	0.7842	0.6237	0.7478	0.5651	0.7594
Wu [10]	0.8551	0.7191	0.8100	0.6776	0.7638
Our method	0.8689	0.7383	0.8152	0.6897	0.7711

**Table 4:** SSIM Comparison of different methods for shaky videos with crowds.

	Video 1	Video 2	Video 3	Video 4	Video 5
Original	0.6964	0.5654	0.6892	0.6716	0.6211
Xu [17]	0.6947	0.5488	0.6775	0.6860	0.6249
Our method + Xu [17]	0.7036	0.5532	0.6858	0.6877	0.6362
Dong [11]	0.7239	0.6316	0.7395	0.7277	0.6918
Wu [10]	0.7652	0.7140	0.7940	0.7731	0.7838
Our method	0.8689	0.7383	0.8152	0.6897	0.7711

**Table 5:** Average SSIM Comparison of different methods in four groups shaky videos.

	Single Object	Only Background	Zooming	Crowds
Original	0.4944	0.6399	0.6163	0.6487
Xu [17]	0.4964	0.6266	0.6289	0.6464
Our method + Xu [17]	0.5587	0.714	0.6642	0.6534
Dong [11]	0.6338	0.7958	0.696	0.7029
Wu [10]	0.7288	0.8685	0.7651	0.766
Our method	0.8041	0.9181	0.8152	0.7896

**Table 6:** SSIM for shaky videos with large foreground.

	Original	Dong [11]	Wu [10]	Our Method
Video 1	0.4288	0.5090	0.5443	0.5566
Video 2	0.2920	0.3703	0.4148	0.4351
Video 3	0.3593	0.4664	0.4998	0.5207
Video 4	0.2403	0.3125	0.3854	0.3978
Video 5	0.4419	0.5705	0.6063	0.6229
Video 6	0.5843	0.6959	0.7164	0.7456
Video 7	0.5867	0.6293	0.6681	0.6702

**Table 7:** SSIM for shaky videos with multi-object.

	Original	Dong [11]	Wu [10]	Our Method
Video 1	0.5349	0.6227	0.6430	0.7588
Video 2	0.4494	0.5835	0.6859	0.8811
Video 3	0.7637	0.8040	0.8486	0.8680
Video 4	0.6441	0.6949	0.7542	0.7972
Video 5	0.5498	0.6494	0.6728	0.7381
Video 6	0.4736	0.5230	0.5432	0.5540

## Data Availability

Not applicable.

## Supplementary Materials

The following are available at https://www.mdpi.com/article/10.3390/s21072505/s1. Video S1: Video stabilization results of different methods.
